# Utilisation of coercive measures in involuntary patients admitted to two psychiatric hospitals in KwaZulu-Natal, South Africa

**DOI:** 10.4102/sajpsychiatry.v32i0.2603

**Published:** 2026-10-23

**Authors:** Sibongile Mashaphu, Zinhle P. Shozi, Vuyokazi Ntlantsana, Shamima Saloojee

**Affiliations:** 1 Discipline of Psychiatry, School of Clinical Medicine, College of Health Sciences, University of KwaZulu-Natal, Durban, South Africa; 2 Department of Psychiatry, Faculty of Health Sciences, Nelson R Mandela School of Medicine, Durban, South Africa

**Keywords:** coercion, involuntary treatment, chemical restraint, mechanical restraint, physical restraint, seclusion

## Abstract

**Background:**

There is a high prevalence of involuntary psychiatric admissions in South Africa, with literature on the measures used to coerce patients into care being limited.

**Aim:**

This study aimed to quantify the coercive measures that were used during involuntary admissions to two psychiatric hospitals in KwaZulu-Natal province, and to identify factors associated with their use.

**Setting:**

King Dinuzulu Hospital Complex in Durban and Townhill Psychiatric Hospital in Pietermaritzburg.

**Methods:**

This was a sub-analysis of a cross-sectional, descriptive, quantitative study of involuntarily admitted patients aged 18 years and above. Data were collected through participant interviews at discharge and by extracting information from patients’ clinical records and *Mental Health Care Act* forms.

**Results:**

Of the 131 patients included, 66% were male, 85.5% were single, and 71.4% were unemployed. The prevalence of chemical, mechanical, physical restraint, and seclusion was 73.3%, 14.5%, 24.43%, and 16.8%, respectively, with 38.2% being exposed to more than one method. Significantly more male patients than female patients were physically restrained h(81% vs 19%; *p* = 0.04), and placed in seclusion (86.4% vs 13.6%; *p* = 0.05). Except for mechanical restraint, a longer length of hospital stay was significantly associated with the other three coercive measures (*p* ≤ 0.05).

**Conclusion:**

Chemical restraint was the most common method of coercion, with more than one-third of patients being exposed to two or more methods.

**Contribution:**

This study highlights the high utilisation of coercive methods and informs the need for reducing their use in psychiatric hospitals in South Africa.

## Introduction

The rate of involuntary psychiatric admissions is increasing globally,^1,2^ with the use of formal coercive measures, such as forced sedation, physical and mechanical restraint, and seclusion being commonly utilised. While the use of some or all of these measures is legally permissible, depending on the countries’ mental health legislation,^3,4,5^ their application is questionable and has generated much ethical debate internationally.^6,7^

In the context of an involuntary hospitalisation on psychiatric grounds, coercion refers to the use of force or duress, or utilising the threat of force to admit and administer treatment to an individual against their will for their own safety or for that of others.^8^ Coercion may occur when individuals experience a loss of control over decisions that they would like to make for themselves through threats, pressure, persuasion, manipulation, or deception on the part of another.^7,9^

Chemical restraint, also known as rapid tranquilisation, is the use of prescribed drugs for sedation in situations where people with mental health disorders present with severe agitation, aggression, or violent behaviours that pose a danger to themselves or others.^10^ Physical restraint is defined as a coercive measure that must be employed as a last resort under any circumstance to ensure the safety of patients by adopting the designed instrument to restrict their physical movement.^11^ Mechanical restraint includes any mechanical device (e.g. the historical straightjacket, the belt with wrist-cuffs, wrist and ankle cuffs tied to the bed, bed-side bars) which, either directly applied to the patient’s body or adjacent to them, is hardly removable and aims to prevent, limit, or control their body movements.^12^ In psychiatry, mechanical restraint is considered complementary and dependent on manual restraint; the latter is usually employed to implement the former, although it may be used alone as in the case of the forced administration of any medication.^12^ However, there are variations in the definitions of various restrictive care practices. A systematic review of 95 studies that provided definitions of different forms of restrictive care practices showed that valid comparisons and reliable measurements of the actual practices are only possible with clear and common definitions of restrictive care practices.^13^ Studies that use broader definitions are more likely to result in a higher measurement outcome than studies that use strict definitions.^13^

It can be argued that there is no justification for the use of any of these measures, as there is no clear evidence of benefit to the individual subjected to them.^14^ There is a real risk of serious physical harm and medical complications, such as venous thromboembolism, cardiac arrest, and pulmonary embolism.^15^

Furthermore, patients who were subjected to these measures viewed them as punitive and felt humiliated,^16^ lonely, and powerless.^17^ However, there is evidence that the use of coercive measures during an involuntary admission is unavoidable and is justifiable to prevent harm to patients and others.^18,19^ Globally, emergency departments and psychiatric facilities experience more violence and aggression than any other healthcare settings.^20^ Therefore, mental health laws globally permit the use of coercive measures to prevent harm to self or others.^18^ The prevalence of the use of coercive measures varies greatly internationally; for example, the use of physical or mechanical restraint in a recent meta-analysis of 77 studies ranged from 0.3% to 54%, with a pooled prevalence of 14.5% for physical restraint, 15.8% for seclusion and 27.7% for chemical restraint reported.^21^

The South African *Mental Health Care Act* (*MHCA*) permits the administration of treatment without consent in involuntary patients, and requires that all institutions with psychiatric admission facilities submit seclusion registers to the designated Mental Health Review Board regularly.^22^ There is no debate that the use of coercive measures needs to be discouraged, restricted, and only used as a last resort to manage behaviour that poses a risk to the patient or others.^5,6,23^ Involuntary psychiatric admissions in KwaZulu-Natal (KZN) province, South Africa, are frequent, with studies indicating that 75% of previous admissions to a psychiatric hospital were either involuntary or assisted,^24^ but with little being known about what measures are used.

The aim of this study was therefore to describe and determine the factors associated with the utilisation of any coercive measures during an involuntary admission to two psychiatric hospitals in KZN, from April 2020 to October 2020.

## Research methods and design

### Study design and procedures

This was a sub-analysis of a cross-sectional, descriptive quantitative study authored by Shozi and Saloojee,^25^ of adult patients who were admitted involuntarily to two psychiatric hospitals in KZN. King Dinuzulu Hospital Complex (KDHC) in eThekwini Municipality (Durban) and Townhill Psychiatric Hospital (THH) in Msunduzi Municipality (Pietermaritzburg) collectively serve as referral hospitals for patients requiring involuntary admission from the entire province of KZN.

The study participants were conveniently recruited over a 6-month study period. Patients were enrolled if they were aged 18 years and older and were admitted involuntarily. Involuntary admissions were according to the specifications of Chapter 5 of *The Act*^26^ and verified by the presence of completed *MHCA* Forms 04, 05, and 07. At the time of discharge, informed consent was obtained from participants, and questionnaires on the admission experience were administered. Apart from the patients’ experience of the involuntary admission, the use of coercive measures during the involuntary admission was also examined. Data were collected at discharge when the patients’ involuntary status had been changed to voluntary. At this point, the patients were freely able to provide voluntary informed consent because they were deemed to be free of psychiatric symptoms. Informed consent was obtained from participants because the information collected utilised rating scales, study-specific questionnaires, patients’ clinical records, treatment scripts and their *MHCA* forms. However, for the purpose of this manuscript, information was extracted only from the patient’s clinical records, treatment scripts and *MHCA* forms.

### Measurements

The socio-demographic and clinical details of participants were extracted from their clinical records, while information regarding involuntary admission was obtained from copies of the (*MHCA*)^22^ Form 04, which is the application to the head of the health establishment for assisted or involuntary admission, and Form 05, which is the report on completion of examination by the mental health practitioner. These forms are routinely filed with the clinical records at the study hospitals, and copies are sent to the mental health review board. Diagnoses were made according to the Diagnostic and Statistical Manual of Mental Disorders, 5th edition, of the American Psychiatric Association (DSM5).^27^ Information regarding chemical restraint was obtained from the patients’ treatment scripts. At the study sites, as part of the standard of care, parenteral sedation is only administered to patients who refuse oral sedation. Further, *pro re nata* (PRN) prescriptions were utilised to collect information regarding medications used to sedate and/or chemically restrain patients as the need arose. Only medications for parenteral sedation were classified as coercive. Long-acting injectable antipsychotic medication and parenteral formulations of oral antipsychotics or benzodiazepines were excluded from the data collected.

In this study, seclusion was defined as the involuntary placement of an individual alone in a locked room,^28^ mechanical restraint as the fixation of at least one of the patient’s limbs by a mechanical appliance (usually cuffed leather restraints, in this study only cuffed leather restraints were utilised),^29^ physical restraint as at least one limb being held down by staff,^30^ and chemical restraint as the administration of parenteral antipsychotic or benzodiazepine medication to control or prevent aggression, anxiety, or violence in people who are likely to cause harm to themselves or others.

### Statistical analysis

Stata version 18 (StataCorp LLC, College Station, TX, US) was used to organise and analyse the data. To determine the utilisation of coercive measures during an involuntary admission, assuming 95% confidence, an acceptable margin of error of 8%, and an assumed prevalence of 30% (given unknown previous prevalence), a minimum sample size of 127 subjects was required. The final sample size was 131, which was increased to reduce type I and type II errors, as well as the effects of known and unknown confounders. Medians and interquartile ranges (IQRs) were used to summarise asymmetrical continuous data (e.g. age and duration of admission in days), and frequencies and percentages were used to summarise categorical data (e.g. race and restraint method used). The chi-squared test was used to test for association between the relevant categorical variables (e.g. restraint method used and gender), and the Wilcoxon rank-sum test was used to compare the relevant continuous (e.g. age) and categorical variables (e.g. restraint method used). Additionally, bivariate analyses were conducted to assess differences in socio-demographic characteristics, clinical features and practices between the two hospitals included in the study.

### Ethical considerations

Ethical clearance to conduct this study was obtained from the University of KwaZulu-Natal Research Ethics Committee (BREC) (Ref. No. BREC/00000208/2019). Gatekeeper permissions were also obtained from the heads of the health establishments at Townhill Hospital and King Dinuzulu Hospital, as well as from the KZN Provincial Department of Health.

## Results

Of the 131 participants included in the study, 71.0% (*n* = 93) were from KDHC, and 29.0% (*n* = 38) from THH. Table 1 presents their socio-demographic and clinical characteristics, with the median age being 33.0 years. Most participants were male, 66% (*n* = 87); single, 85.4% (*n* = 112); unemployed, 77.9% (*n* = 102); had at least a high school level of education, 54.2% (*n* = 71); and used substances, 75.6% (*n* = 99). The most common diagnosis was a psychotic disorder (79.4%, *n* = 104), and the average duration of hospital stay was 17.0 days. Regarding the reasons for admission, 43.1% (*n* = 56) were admitted for treatment of acute symptoms, 37.7 % (*n* = 49) posed a danger to others, 15.4% (*n* = 20) posed a danger to themselves, and 3.9 % (*n* = 5) had destroyed property.

**TABLE 1:** Socio-demographic and clinical variables (*N* = 131).

Variable	*n*	%	Median	IQR
**Age**	-	-	27.0	24.0, 33.0
**Gender**				
Female	44	33.6	-	-
Male	87	66.4	-	-
**Race**				
Black people	102	77.9	-	-
Coloured people	11	8.4	-	-
White people	5	3.8	-	-
Indian people	13	9.9	-	-
**Home language**				
IsiZulu	94	71.8	-	-
English	27	20.6	-	-
Afrikaans	2	1.5	-	-
Other	8	6.1	-	-
**Marital status†**				
Single	112	86.2	-	-
Married	10	7.7	-	-
Divorced	7	5.4	-	-
Cohabiting	1	0.8	-	-
**Type of housing**				
Informal	17	13.0	-	-
Formal	114	87.0	-	-
**Matric**				
Below matric	60	45.8	-	-
Matric and above	71	54.2	-	-
**Disability grant**				
No	107	81.7	-	-
Yes	24	18.3	-	-
**Employed**				
No	102	77.9	-	-
Yes	29	22.1	-	-
**History of arrest**				
No	103	78.6	-	-
Yes	28	21.4	-	-
**Use of substances?**				
No	32	24.4	-	-
Yes	99	75.6	-	-
**Type of substance used†**				
Tobacco	20	20.0	-	-
Alcohol	14	14.0	-	-
Cannabis	32	33.0	-	-
Other	32	33.0		
**Diagnostic categories**			-	-
Schizophrenia and related disorders	104	79.4	-	-
Mood disorders	14	10.7	-	-
Other	13	9.9	-	-
Duration of admission (days)	-	-	17.0	9.0, 69.0

IQR, interquartile range.

† , Data were missing for some participants.

Table 2 shows that 38.2 % (*n* = 50) of the participants were exposed to two or more methods of coercion, and 3.1 % (*n* = 4) were exposed to all four methods. Chemical restraint was used for 73.3% (*n* = 96), mechanical restraint for 14.5% (*n* = 19), physical restraint for 24.43% (*n* = 32), and 16.8% (*n* = 22) were secluded.

**TABLE 2:** Methods of coercion and reasons for admission.

Variables	*n*	%	Median	IQR
**Reason for admission**	-	-	-	-
Danger to others	49	37.69	-	-
Danger to self	20	15.38	-	-
Destruction of property	5	3.85	-	-
Acute mania	15	11.54	-	-
Acute psychosis	41	31.54	-	-
**Number of coercive measures used**	-	-	1	1–2
0	32	24.43	-	-
1	49	37.40	-	-
2	34	25.95	-	-
3	12	9.16	-	-
4	4	3.05	-	-
**Chemical restraint used during this admission**	-	-	-	-
No	35	26.7	-	-
Yes	96	73.3	-	-
**Mechanical restraint**	-	-	-	-
No	112	85.5	-	-
Yes	19	14.5	-	-
**Physical restraint used during current admission**	-	-	-	-
No	99	75.6	-	-
Yes	32	24.4	-	-
**Seclusion used during this admission**	-	-	-	-
No	109	83.2	-	-
Yes	22	16.8	-	-

IQR, interquartile range.

Table 3 shows the association between socio-demographic and clinical variables and the use of various methods of coercion. There was a significant association between gender and the use of coercive measures, with more males than females being physically restrained (81% vs 19%; *p* = 0.04), and placed in seclusion (86.4% vs 13.6%; *p* = 0.05). A longer hospital stay was significantly associated with physical restraint, chemical restraint and seclusion (*p* ≤ 0.05), but not with mechanical restraint (*p* = 0.09). There was a significant association between seclusion and those who were arrested by police previously (*p* = 0.044) and diagnosed with a psychotic disorder (*p* = 0.037).

**TABLE 3:** Association between socio-demographic and clinical variables with injury and coercive measures.

Variable	Injury								*p*	Chemical restraint								*p*	Mechanical restraint								*p*	Physical restraint								*p*	Seclusion								*p*
	No				Yes					No				Yes					No				Yes					No				Yes					No				Yes				
	*n*	%	Median	IQR	*n*	%	Median	IQR		*n*	%	Median	IQR	*n*	%	Median	IQR		*n*	%	Median	IQR	*n*	%	Median	IQR		*n*	%	Median	IQR	*n*	%	Median	IQR		*n*	%	Median	IQR	*n*	%	Median	IQR	
***N***	108	23	-	-	-	-	-	-		35	96	-	-	-	-	-	-		112	19	-	-	-	-	-	-		99	32	-	-	-	-	-	-		109	22	-	-	-	-	-	-	
**Age**	-	-	27.0	23.5, 34.5	-	-	29.5	24.0, 32.0	0.76	-	-	25.5	20.5, 31.5			28.0	24.0, 33.0	0.19			27.0	23.0, 33.0			28.0	25.0, 35.0	0.34			27.0	24.0, 33.0			26.0	24.0, 31.0	0.82			27.0	24.0, 35.0			24.0	24.0, 28.0	0.31
**Gender**	-	-	-	-	-	-	-	-	0.40	-	-	-	-	-	-	-	-	0.75	-	-	-	-	-	-	-	-	0.11	-	-	-	-	-	-	-	-	0.041	-	-	-	-	-	-	-	-	0.046
Female	38	35.2	-	-	6	26.1	-	-	-	11	31	-	-	33	34	-	-	-	41	36.6	-	-	3	15.8	-	-	-	-	38	-	-	6	19	-	-	-	41	37.6	-	-	3	13.6	-	-	-
Male	70	64.8	-	-	17	73.9				24	69	-	-	63	66	-	-	-	71	63.4	-	-	16	84.2	-	-	-	61	62	-	-	26	81	-	-	-	68	62.4	-	-	19	86.4	-	-	-
**Race**	-	-	-	-	-	-	-	-	0.62	-	-	-	-	-	-	-	-	0.42	-	-	-	-	-	-	-	-	0.087	-	-	-	-	-	-	-	-	0.66	-	-	-	-	-	-	-	-	0.30
Black people	84	77.8	-	-	18	78.3	-	-	-	26	74	-	-	76	79	-	-	-	90	80.4	-	-	12	63.2	-	-	-	76	77	-	-	26	81	-	-	-	82	75.2	-	-	20	90.9	-	-	-
Coloured people	8	7.4	-	-	3	13.0	-	-	-	2	6	-	-	9	9	-	-	-	7	6.2	-	-	4	21.1	-	-	-	10	10	-	-	1	3	-	-	-	9	8.3	-	-	2	9.1	-	-	-
White people	4	3.7	-	-	1	4.3	-	-	-	1	3	-	-	4	4	-	-	-	5	4.5	-	-	0	0.0	-	-	-	4	4	-	-	1	3	-	-	-	5	4.6	-	-	0	0.0	-	-	-
Indian people	12	11.1	-	-	1	4.3	-	-	-	6	17	-	-	7	7	-	-	-	10	8.9	-	-	3	15.8	-	-	-	9	9	-	-	4	12	-	-	-	13	11.9	-	-	0	0.0	-	-	-
**Home language**	-	-	-	-	-	-	-	-	1.00	-	-	-	-	-	-	-	-	0.63	-	-	-	-	-	-	-	-	0.28	-	-	-	-	-	-	-	-	0.87	-	-	-	-	-	-	-	-	0.39
IsiZulu	77	71.3	-	-	17	73.9	-	-	-	24	69	-	-	70	73	-	-	-	83	74.1	-	-	11	57.9	-	-	-	69	70	-	-	25	78	-	-	-	75	68.8	-	-	19	86.4	-	-	-
English	22	20.4	-	-	5	21.7	-	-	-	7	20	-	-	20	21	-	-	-	20	17.9	-	-	7	36.8	-	-	-	21	21	-	-	6	19	-	-	-	25	22.9	-	-	2	9.1	-	-	-
Afrikaans	2	1.9	-	-	0	0.0	-	-	-	1	3	-	-	1	1	-	-	-	2	1.8	-	-	0	0.0	-	-	-	2	2	-	-	0	0	-	-	-	2	1.8	-	-	0	0.0	-	-	-
Other	7	6.5	-	-	1	4.3	-	-	-	3	9	-	-	5	5	-	-	-	7	6.2	-	-	1	5.3	-	-	-	7	7	-	-	1	3	-	-	-	7	6.4	-	-	1	4.5	-	-	-
**Marital status**	-	-	-	-	-	-	-	-	0.67	-	-	-	-	-	-	-	-	0.27	-	-	-	-	-	-	-	-	0.23	-	-	-	-	-	-	-	-	0.69	-	-	-	-	-	-	-	-	0.47
Single	91	85.0	-	-	21	91.3	-	-	-	28	82	-	-	84	88	-	-	-	98	88.3	-	-	14	73.7	-	-	-	82	84	-	-	30	94	-	-	-	91	84.3	-	-	21	95.5	-	-	-
Married	8	7.5	-	-	2	8.7	-	-	-	5	15	-	-	5	5	-	-	-	7	6.3	-	-	3	15.8	-	-	-	9	9	-	-	1	3	-	-	-	10	9.3	-	-	0	0.0	-	-	-
Divorced	7	6.5	-	-	0	0.0	-	-	-	1	3	-	-	6	6	-	-	-	5	4.5	-	-	2	10.5	-	-	-	6	6	-	-	1	3	-	-	-	6	5.6	-	-	1	4.5	-	-	-
Cohabiting	1	0.9	-	-	0	0.0	-	-	-	0	0	-	-	1	1	-	-	-	1	0.9	-	-	0	0.0	-	-	-	1	1	-	-	0	0	-	-	-	1	0.9	-	-	0	0.0	-	-	-
**Type of housing**	-	-	-	-	-	-	-	-	0.039	-	-	-	-	-	-	-	-	0.75	-	-	-	-	-	-	-	-	0.27	-	-	-	-	-	-	-	-	0.003	-	-	-	-	-	-	-	-	0.43
Informal	11	10.2	-	-	6	26.1	-	-	-	4	11	-	-	13	14	-	-	-	13	11.6			4	21.1	-	-	-	8	8	-	-	9	28	-	-	-	13	11.9	-	-	4	18.2	-	-	-
Formal	97	89.8	-	-	17	73.9	-	-	-	31	89	-	-	83	86	-	-	-	99	88.4			15	78.9	-	-	-	91	92	-	-	23	72	-	-	-	96	88.1	-	-	18	81.8	-	-	-
**Level of education**	-	-	-	-	-	-	-	-	< 0.001	-	-	-	-	-	-	-	-	0.70	-	-	-	-	-	-	-	-	0.10	-	-	-	-	-	-	-	-	0.79	-	-	-	-	-	-	-	-	0.066
Below matric	42	38.9	-	-	18	78.3	-	-	-	17	49	-	-	43	45	-	-	-	48	42.9	-	-	12	63.2	-	-	-	46	46	-	-	14	44	-	-	-	46	42.2	-	-	14	63.6	-	-	-
Matric or higher	66	61.1	-	-	5	21.7	-	-	-	18	51	-	-	53	55	-	-	-	64	57.1	-	-	7	36.8	-	-	-	53	54	-	-	18	56	-	-	-	63	57.8	-	-	8	36.4	-	-	-
**Disability grant**	-	-	-	-	-	-	-	-	0.57	-	-	-	-	-	-	-	-	0.42	-	-	-	-	-	-	-	-	0.11	-	-	-	-	-	-	-	-	0.55	-	-	-	-	-	-	-	-	0.23
No	87	80.6	-	-	20	87.0	-	-	-	27	77	-	-	80	83	-	-	-	94	83.9	-	-	13	68.4	-	-	-	82	83	-	-	25	78	-	-	-	91	83.5	-	-	16	72.7	-	-	-
Yes	21	19.4	-	-	3	13.0	-	-	-	8	23	-	-	16	17	-	-	-	18	16.1	-	-	6	31.6	-	-	-	17	17	-	-	7	22	-	-	-	18	16.5	-	-	6	27.3	-	-	-
**Employed**	-	-	-	-	-	-	-	-	0.29	-	-	-	-	-	-	-	-	0.72	-	-	-	-	-	-	-	-	0.28	-	-	-	-	-	-	-	-	0.97	-	-	-	-	-	-	-	-	0.78
No	86	79.6	-	-	16	69.6	-	-	-	28	80	-	-	74	77	-	-	-	89	79.5	-	-	13	68.4	-	-	-	77	78	-	-	25	78	-	-	-	84	77.1	-	-	18	81.8	-	-	-
Yes	22	20.4	-	-	7	30.4	-	-	-	7	20	-	-	22	23	-	-	-	23	20.5	-	-	6	31.6	-	-	-	22	22	-	-	7	22	-	-	-	25	22.9	-	-	4	18.2	-	-	-
**History of arrest**	-	-	-	-	-	-	-	-	0.24	-	-	-	-	-	-	-	-	0.23	-	-	-	-	-	-	-	-	0.24	-	-	-	-	-	-	-	-	0.68	-	-	-	-	-	-	-	-	0.044
No	87	80.6	-	-	16	69.6	-	-	-	25	71	-	-	78	81	-	-	-	90	80.4	-	-	13	68.4	-	-	-	77	78	-	-	26	81	-	-	-	82	75.2	-	-	21	95.5	-	-	-
Yes	21	19.4	-	-	7	30.4	-	-	-	10	29	-	-	18	19	-	-	-	22	19.6	-	-	6	31.6	-	-	-	22	22	-	-	6	19	-	-	-	27	24.8	-	-	1	4.5	-	-	-
**Use of substances**	-	-	-	-	-	-	-	-	0.063	-	-	-	-	-	-	-	-	0.24	-	-	-	-	-	-	-	-	1.00	-	-	-	-	-	-	-	-	0.93	-	-	-	-	-	-	-	-	1.00
No	30	27.8	-	-	2	8.7	-	-	-	6	17	-	-	26	27	-	-	-	28	25.0	-	-	4	21.1	-	-	-	24	24	-	-	8	25	-	-	-	27	24.8	-	-	5	22.7	-	-	-
Yes	78	72.2	-	-	21	91.3	-	-	-	29	83	-	-	70	73	-	-	-	84	75.0	-	-	15	78.9	-	-	-	75	76	-	-	24	75	-	-	-	82	75.2	-	-	17	77.3	-	-	-
**Type of substance**	-	-	-	-	-	-	-	-	0.084	-	-	-	-	-	-	-	-	0.83	-	-	-	-	-	-	-	-	0.11	-	-	-	-	-	-	-	-	0.089	-	-	-	-	-	-	-	-	0.71
Tobacco	19	25	-	-	1	5	-	-	-	7	24	-	-	13	19	-	-	-	16	19			4	27	-	-	-	17	23	-	-	3	13	-	-	-	18	22	-	-	2	12	-	-	-
Alcohol	11	14	-	-	3	14	-	-	-	5	17	-	-	9	13	-	-	-	14	17			0	0	-	-	-	10	13	-	-	4	17	-	-	-	11	14	-	-	3	18	-	-	-
Cannabis	26	34	-	-	6	29	-	-	-	9	31	-	-	23	33	-	-	-	29	35			3	20	-	-	-	28	37	-	-	4	17	-	-	-	25	31	-	-	7	41	-	-	-
Other	21	27	-	-	11	52	-	-	-	8	28	-	-	24	35	-	-	-	24	29			8	53	-	-	-	20	27	-	-	12	52	-	-	-	27	33	-	-	5	29	-	-	-
**Diagnosis**	-	-	-	-	-	-	-	-	0.28	-	-	-	-	-	-	-	-	0.23	-	-	-	-	-	-	-	-	1.00	-	-	-	-	-	-	-	-	0.63	-	-	-	-	-	-	-	-	0.037
Psychotic d/o	86	79.6	-	-	18	78.3	-	-	-	25	71	-	-	79	82	-	-	-	89	79.5	-	-	15	78.9	-	-	-	77	78	-	-	27	84	-	-	-	82	75.2	-	-	22	100.0	-	-	-
Mood d/o	13	12.0	-	-	1	4.3	-	-	-	4	11	-	-	10	10	-	-	-	12	10.7	-	-	2	10.5	-	-	-	12	12	-	-	2	6	-	-	-	14	12.8	-	-	0	0.0	-	-	-
Other	9	8.3	-	-	4	17.4	-	-	-	6	17	-	-	7	7	-	-	-	11	9.8	-	-	2	10.5	-	-	-	10	10	-	-	3	9	-	-	-	13	11.9	-	-	0	0.0	-	-	-
**Duration of admission (days)**	-	-	17.0	9.0, 52.0	-	-	16.0	8.0, 94.0	0.83			12.0	6.0, 34.0			21.5	9.0, 79.5	0.054			15.0	9.0, 40.5			56.0	8.0, 118.0	0.087			14.0	8.0, 42.0			36.0	11.0, 99.5	0.025			13.0	7.0, 34.0			79.5	37.0, 201.0	< 0.001

IQR, interquartile range.

*p, p*-value.

Participants in the two hospitals were similar in their socio-demographic and clinical characteristics, except for duration of admission and seclusion. The median duration of admission was significantly longer in THH (100 days, IQR 77–213 days) than in KDHC (11 days, IQR 7–22). Seclusion during current admission was more likely in THH, where 32% (*n* = 12) of participants were secluded, compared with 11% (*n* = 10) at KDHC (*p* = 0.004).

## Discussion

In a study sample of people who were admitted involuntarily, this study shows that most participants were single (85.4%), unemployed (71.4%), males (66%), used substances (75.6%), and were diagnosed with a disorder from the schizophrenia and related disorders section of the DSM 5 (79.4%). About three out of every four participants (73%) were exposed to chemical restraint, and more than a third were subjected to two or more methods of coercion. A longer duration of admission was significantly associated with chemical restraint, physical restraint, and seclusion, with males being more likely to be placed in seclusion than females.

### Chemical restraint

The prevalence of chemical restraint (73%) in this study is much higher than that reported in two international meta-analyses. The prevalence is more than double that reported in the Belayneh et al. (2025)^31^ meta-analysis (26%), and higher than that in the Muir-Cochrane et al.’s^32^ meta-analysis (43%). Our prevalence cannot be directly compared with other studies because, as highlighted in the literature, there is significant variation in the prevalence of chemical restraint due to differences in its definition and the methods used to investigate the prevalence of coercive measures.^31^ For example, the European Evaluation of Coercion in Psychiatry and Harmonization of Best Clinical Practice (EUNOMIA) Project across 10 European countries reported that the proportion of participants who were exposed to chemical restraint in psychiatric inpatient facilities ranged from 31% in Greece to 81% in Sweden.^5^ It is possible that the high rate of chemical restraint in this study may be explained by: (1) the inclusion of *pro re nata* (PRN) administration of parenteral antipsychotic medication in patients who refused oral antipsychotics at any time during their admission, (2) nursing staff who may have over-utilised chemical restraint because they wanted to prevent rather than contain aggressive incidents and (3) we used a wide definition of chemical restraint, that is, we included any patient who was sedated parenterally in the definition of chemical restraint.

### Physical restraint

The prevalence of physical restraint in this study (24%) was also higher than the meta-analytic prevalence of (14%).^21^ A likely explanation for this higher figure is its frequent use as a temporary measure for administering other medications. At the study sites, it is common practice for uniformed hospital security guards to be summoned to ‘convince’ patients to accept sedation or assist with physical restraint if they continue to refuse medication.

### Mechanical restraint

Mechanical restraint in this study was the least commonly used coercive measure (14%), the same as in a study from Italy (14%).^33^ However, a recent meta-analysis confirmed that, similar to the wide variation in prevalence of other coercive measures, the figures between and within countries varied from < 1% to 51% globally.^34^ Information from Africa is sparse, with a study from a general hospital in Cape Town reporting no mechanical restraint in the psychiatric wards, with mechanical restraint being used more frequently in medically ill and elderly patients, as their over-sedation can lead to falls and injuries in the latter.^35^ A systematic review reported that there was insufficient evidence to conclude that the outcomes of mechanical restraint were more effective than seclusion.^36^ Mechanical restraint may have been used less frequently than the other coercive measures due to the younger age of the participants included in this study, the extensive use of chemical restraint, and the availability of multiple seclusion rooms in the psychiatric hospitals.

### Seclusion

Similar to findings on physical restraint, this study found that the prevalence of seclusion (16.8 %) was consistent with the findings by Belayneh et al.’s meta-analysis (15.8%).^21,37^ The prevalence in this study is higher than that reported in a study from Zimbabwe (8.5%),^38^ but is almost half that reported in a study from Malawi (30.3%).^39^ Two studies from South Africa, one from Cape Town^40^ and the other from Gauteng province,^41^ reported on the use of seclusion in psychiatric hospitals but did not indicate the prevalence. In our study, all the secluded patients had a diagnosis of a psychotic disorder, with a study from Geneva showing higher seclusion rates for patients with psychotic disorders (53.7%) compared with bipolar disorders (28.1%).^42^

### Factors influencing the implementation of restrictive care practices

Psychiatric institutions in KZN have unique challenges that include poorly staffed wards, a shortage of inpatient psychiatric beds, inadequate seclusion facilities, human rights abuses and long duration of hospital stays.^43,44^ However, the reasons for the differences in duration of admission and seclusion practices between the hospitals were unclear. This may partly reflect differences in service demand, bed pressure and patient flow. The population served by KDHC in eThekwini Municipality is more than six times that of Msunduzi Municipality served by THH.^45^ Seclusion practices could be due to differences in institutional culture and practices, differences in the clinical profiles of patients, or differences in documentation practices between the two facilities. The decision to sedate a patient on admission also has multiple consequences, including implications for the outcome of the admission decision itself, which may be rationalised by the fact that the individual has been sedated.^46^

### Socio-demographic and clinical associations with exposure to coercive measures

Our finding of an association between gender and seclusion is consistent with the literature, with men significantly more likely to be secluded than women, as reported in studies across Europe and Australia (52% – 56%).^40,47,48^ A longer length of stay was associated with three of the four coercive methods in this study (*p* < 0.001). Other studies that examined the overall use of coercive measures in different settings found a longer length of stay to be associated with higher utilisation of seclusion.^49,50,51,52^

### Limitations

The study has several limitations, including a cross-sectional design, a convenience sampling strategy, and a small sample size. Further, we did not collect information on the types and doses of medications used during chemical restraint and seclusion; we did not review seclusion registers according to the requirements of the *MHCA* at the study sites, and we did not confirm that patients refused oral medications by examining nursing records. This study also relied on the documentation of coercive measures in the hospital records, which may have been incomplete. Information from a single province limits the generalisability of the findings to other provinces in South Africa.

## Conclusion

The findings of this study show that in a group of mostly young males with psychotic disorders, three-quarters were subject to forced medication during their involuntary admission. Physical restraint and seclusion were less common, but taken together, the high prevalence of coercive measures in a population of patients who were admitted involuntarily in this study is of concern. Despite substantial research on coercive practices that has emerged in the past 20 years, our understanding of patients’ perception of coercion and its opposite, patients’ perception of autonomy and empowerment, is still limited. There are multiple reports of the lack of justification for the use of coercion in psychiatry,^53,54^ but up until now there has been a lack of policy development to promote preventive measures which are suited to reduce the number of compulsory admissions and to promote patients’ autonomy. This highlights the need for studies investigating coercive measures and any noticeable efforts to reduce this practice within our mental health institutions by introducing more focused training for nursing staff, employment of dedicated security guards stationed in psychiatric wards, and an improvement in the number and structure of psychiatric inpatient facilities in KZN.
